# Intrahepatic Gallbladder Rupture and Biloma Mimicking Pulmonary Embolism Following Orthopedic Surgery

**DOI:** 10.7759/cureus.46905

**Published:** 2023-10-12

**Authors:** Christos Doudakmanis, George Dimeas, Ilias E Dimeas, Maria M Pitsilka, Zoe Daniil

**Affiliations:** 1 Department of Critical Care Medicine, University Hospital of Larissa, Larissa, GRC; 2 2nd Propaedeutic Department of Surgery, Laiko General Hospital of Athens, Athens, GRC; 3 Department of Respiratory Medicine, University Hospital of Larissa, Larissa, GRC

**Keywords:** pulmonary embolism, sphincter of oddi, epidural morphine, complication, postoperative, rupture, gallbladder, intrahepatic, pulmonary embolism (pe), biloma

## Abstract

In this case report, a 75-year-old male with a history of coronary artery disease, type 2 diabetes, hypertension, and benign prostate hypertrophy developed postoperative fever and chest pain following left knee arthroplasty. Upon admission to the emergency department, pulmonary embolism was considered highly probable, and the patient was treated with anticoagulation and antibiotics due to diagnostic uncertainty. However, further investigations revealed a complex condition involving an intraparenchymal gallbladder rupture resulting in a biloma secondary to choledocholithiasis. The patient's history of receiving spinal anesthesia with intrathecal morphine was identified as a potential causative factor to the sphincter of Oddi constriction, leading to increased biliary pressure and gallbladder rupture. This case highlights the importance of having a broad differential diagnosis in postoperative patients, especially when the clinical presentation is atypical. With the diagnosis being confirmed, the patient underwent successful treatment, including biliary stenting, drainage of the biloma, and ultimately cholecystectomy. This case underlines the need for vigilance and a multidisciplinary approach in managing complex postoperative complications, emphasizing that clinical presentations may sometimes deviate significantly from the expected, requiring further investigation and individualized treatment.

## Introduction

Chest pain is among the most common causes of admission to the emergency department. Due to its heterogeneous clinical presentation, the differential diagnosis is broad and includes both thoracic disorders and abdominal diseases, as even myocardial infarction or pulmonary embolism (PE) can be presented as abdominal pain. Most common are pneumonia, pneumothorax, acute coronary syndrome, or acute pericarditis presenting also with dyspnea and chest pain [1]. Most importantly, after thorough examination and evaluation, a cardiovascular cause should be ruled out first. Subsequently, the differential diagnosis should include pneumonia and inflammatory and musculoskeletal causes [2].

PE is a life-threatening condition and is a high-yield diagnosis. This clinical entity occurs when a blood clot, coming through the venous flow, obstructs the blood supply of the lungs. Clinical manifestations are not specific, making the diagnosis hard for the physicians. Patients with mild cases can be asymptomatic or be presented only with mild dyspnea and dizziness. The most common associated symptoms are chest pain, especially pleuritic chest pain, difficulty breathing at rest, and hemoptysis. In worst cases, patients develop respiratory failure, hemodynamic instability, and acute heart failure leading to death before even arriving at the hospital [3]. For clinical evaluation, the Wells score and revised Geneva score are mainly used to categorize the patient's probability of PE as low, intermediate, or high in addition to D-dimer testing for the final assessment of the imaging necessity. Currently, computed tomography pulmonary angiogram (CTPA) is the reference standard in the diagnosis of PE due to its high accuracy and easy availability [4]. Abdominal pain due to PE is not usual but still exists, leading to other diagnostic thoughts of diseases in the biliary system or pancreatitis [5].

Besides idiopathic forms of PE and those related to patients’ eigen factors, trauma and immobilization during the postoperative course of a patient increase the incidence risk [6,7].

Gallbladder rupture is a clinical event that more often happens in cases of complicated acute cholecystitis. However, in rare cases, it may be present even without inflammation and is characterized as spontaneous gallbladder rupture. Patients presenting with spontaneous gallbladder rupture often have chronic alterations, which contribute to the event. Chronic inflammation and chronic ischemic alterations are the most important causative factors in these cases [8,9].

Bilomas are collections of bile outside the bile ducts, and in most cases, they are associated with iatrogenic or traumatic disruption of the continuity of the biliary tree. Rarely, in the absence of the causes mentioned above, spontaneous bilomas may occur. They come as a result of an obstruction in the biliary tree, such as in the case of choledocholithiasis, and a sudden increase in the intrabiliary pressure [10].

## Case presentation

**Figure 1:**
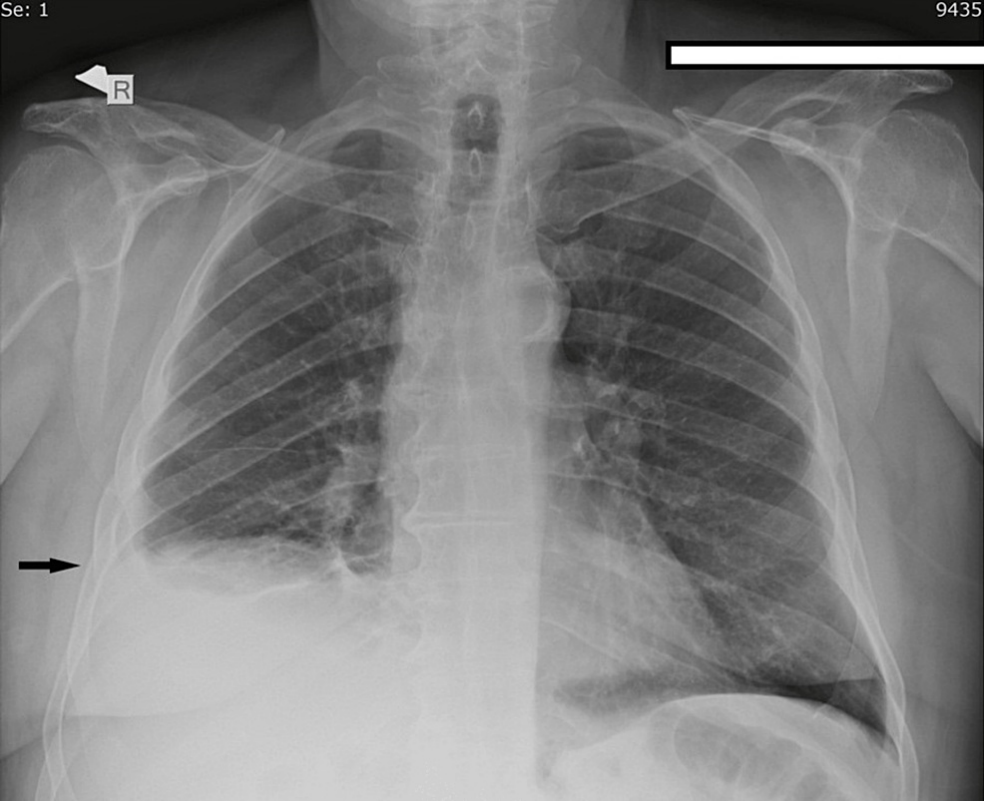
Chest X-ray on admission Elevation of right hemidiaphragm and blunting of right costophrenic angle.

Our patient is a 75-year-old male with a medical history of coronary disease, type 2 diabetes, hypertension, and benign prostate hypertrophy. The patient presented to the emergency department (ED) due to chest pain located in the right hemithorax, deteriorating with inspiratory movements, and reported a fever of up to 39°C. All the symptoms had started a few hours before the patient's admission to the ED. The patient had undergone total left knee arthroplasty five days prior to admission to the ED without any postoperative complications and he was receiving low molecular weight heparin in prophylactic dosing, in addition to the single antiplatelet factor for the coronary artery disease. During clinical assessment, lowered breathing sounds were noted in the right hemithorax, and chest X-ray showed blunting in the right costophrenic angle (Figure 1). The patient was mild tachycardic with 105 beats per minute sinus rhythm on an electrocardiogram. No pathological heart sounds were detected and no other abnormalities were found in the clinical examination. Blood examination showed a mild normocytic anemia, a mild elevated C-reactive protein, an erythrocyte sedimentation rate value of 50 mm/hour (normal value: <37.5 mm/hour), and a positive D-dimer test. Positive D-dimer examination was an expected postoperative result but was asked to rule out pulmonary thromboembolism. Due to high suspicion of PE with a Wells score of 6 points (tachycardia = 1.5 points, PE #1 diagnosis or equally likely = 3 points, surgery in the previous four weeks = 1.5 points), he was admitted to the department of respiratory medicine, where he received intravenous hydration and broad-spectrum antibiotics, as in case of lower respiratory tract infection, and therapeutic dosage of low molecular weight heparin, as in case of PE.

**Figure 2:**
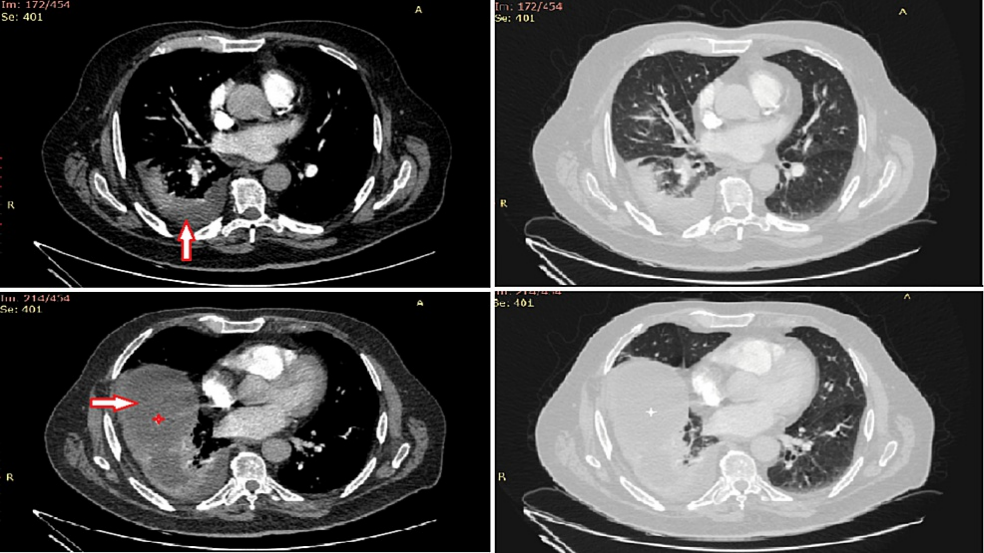
Computed tomography pulmonary angiogram images The arrow points to the right pleural effusion and partial atelectasis of the right lower lobe. In the lower segments of the scan, the subcapsular liver lesion is marked with a star.

Having taken into consideration a prior surgical intervention with foreign body placement, we had an X-ray of the knee, and it was examined by an orthopedic surgeon. There were no signs of inflammation to the operated joint, that could justify the fever. Initial evaluation ruled out other causes of fever, like urinary tract infections and thrombophlebitis. Due to a history of severe allergic reactions to intravenous contrast agents, due to hospital protocol, the patient had to receive premedication with corticosteroids and antihistamines before undergoing a CT of pulmonary arteries to count out PE as a probable cause for his symptoms. Unfortunately, ventilation/perfusion scan or magnetic resonance angiography as alternatives are not available in our area. Two days later, he finally had a CTPA, which was negative for PE. However, the scan showed a pleural effusion in the right hemithorax, alongside an atelectasis. In addition, it was noted that the right hemidiaphragm was elevated and underneath there was a subcapsular liver lesion, which was in contact with the gallbladder (Figure 2). The attending physicians discontinued anticoagulation therapy, as the incidence of a spontaneous subcapsular liver hematoma was considered possible, although there was no drop in the hemoglobin in the blood tests.

**Figure 3:**
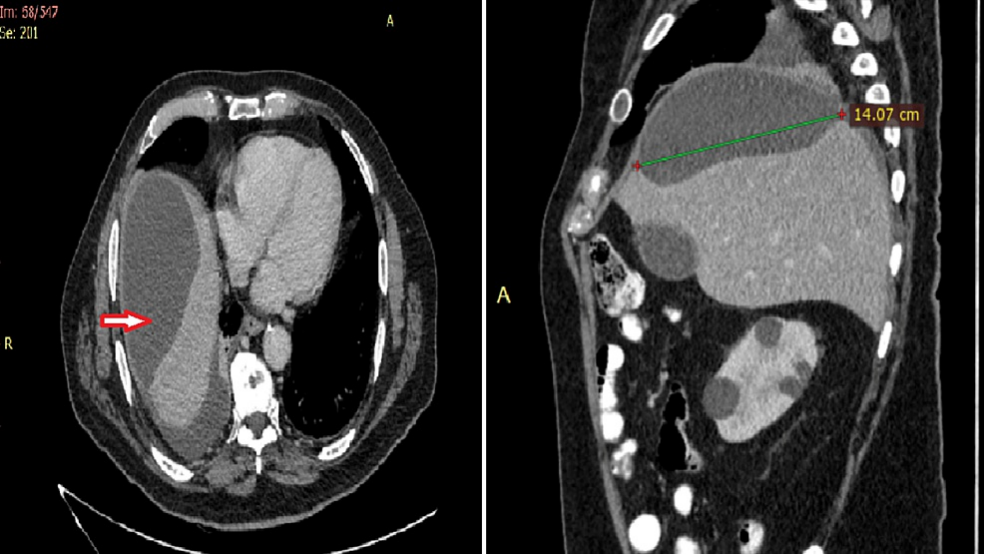
Abdominal CT images The arrow shows the subcapsular liver lesion in the sagittal plane. Its diameter is measured at about 14 cm.

**Figure 4:**
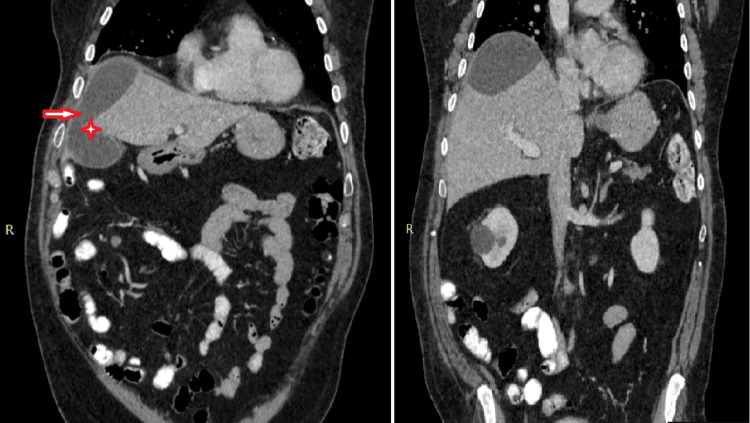
Coronal plane of the abdominal CT The star highlights the continuity between the gallbladder and the subcapsular lesion.

To further investigate the subcapsular lesion, a CT of the abdomen was requested. It was revealed that the subcapsular lesion, measuring 14.07 x 7.5 cm, was in contact with a discontinuation of the gallbladder wall in the anatomic place of the fundus. The gallbladder was full of lithiasis, but without any radiological sign of inflammation (Figures 3, 4).

**Figure 5:**
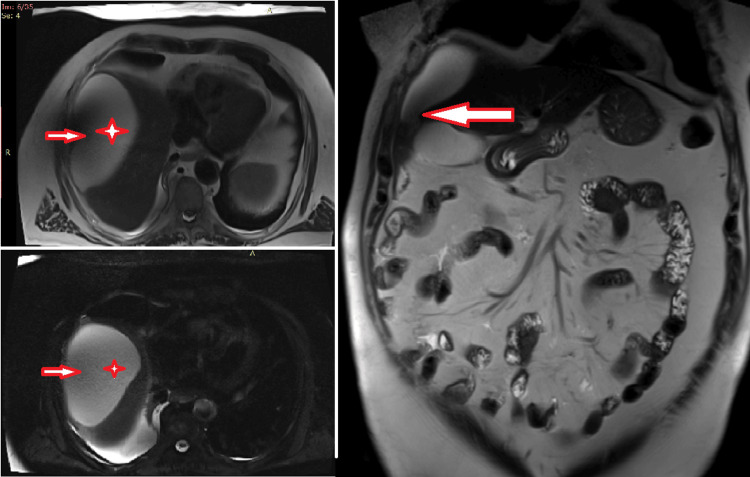
Magnetic resonance cholangiopancreatography The star depicts the fluid collection and the arrow points out the connection between the gallbladder and the subcapsular fluid collection.

Following these results, the patient was transferred to the department of surgery for further investigation and treatment. A subsequent magnetic resonance cholangiopancreatography (MRCP) showed mild intrahepatic biliary dilation with a dilated common bile duct of at least 8 mm. There were also multiple defects in the common bile duct as in the case of choledocholithiasis. This scan confirmed the multiple lithiasis of the gallbladder, but it also revealed its connection with a subcapsular fluid collection, located in the anterolateral segments of the right hepatic lobe, suggesting the diagnosis of biloma, following an intraparenchymal gallbladder rupture (Figure 5).

Given these results, an endoscopic retrograde cholangiopancreatography (ERCP) was performed (five days after admission). Sphincterotomy was performed and a 10-French (diameter) 10 cm (length) plastic stent was placed in the common bile duct. Gastroenterologists recommended that surgical intervention to drain the biloma should be prioritized and common bile duct cannulation to remove the gallstones could be done afterwards.

**Figure 6:**
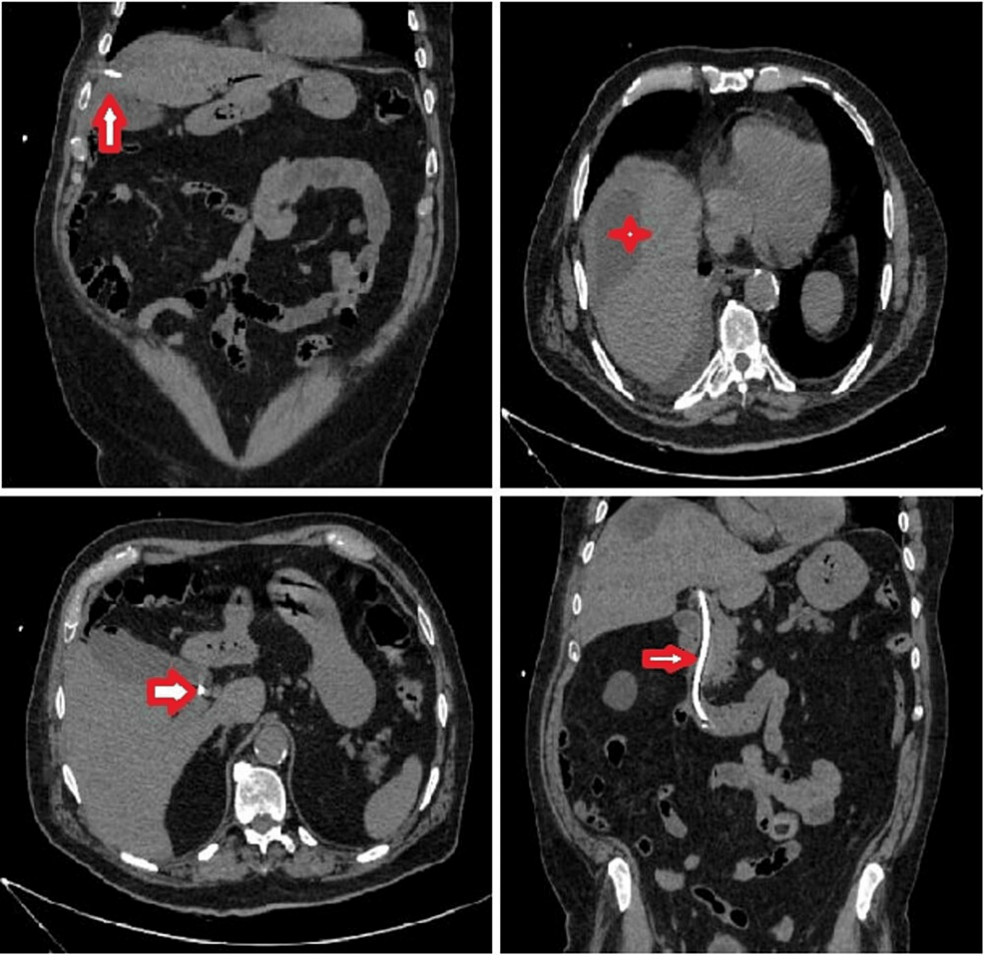
Images of the abdominal CT following percutaneous drainage of the collection and common bile duct stenting The white arrow points to the drain, the star represents the fluid collection smaller in size, and the arrow with blacked margins points to the stent within the common bile duct.

Afterward, the biloma was drained under CT guidance by interventional radiologists, and a drain tube was placed. The drain had a mixed texture of bile with gallbladder sludge. Several days after, a CT imaging showed that the fluid collection was diminished in size and quantity (Figure 6). Following this finding, on the 12th day of the hospitalization, the patient underwent open cholecystectomy through a Kocher incision, the residual biloma was drained effectively, and two drain tubes were placed on the site of interest. Intraoperatively, the gallbladder had a few soft adhesions with the major omentum, the cholecystectomy took place without any major difficulties, and the surgical team found out that the biloma had turned into an abscess. Following the surgery, another ERCP took place, and a stent was placed in the common bile duct. The postoperative course was uneventful, and the patient was discharged a few days later. Biopsy results showed macroscopically a gallbladder full of gallstones and microscopically, it showed alterations compatible with chronic cholecystitis, without signs of acute inflammation within the gallbladder walls, which were described as thin.

**Figure 7:**
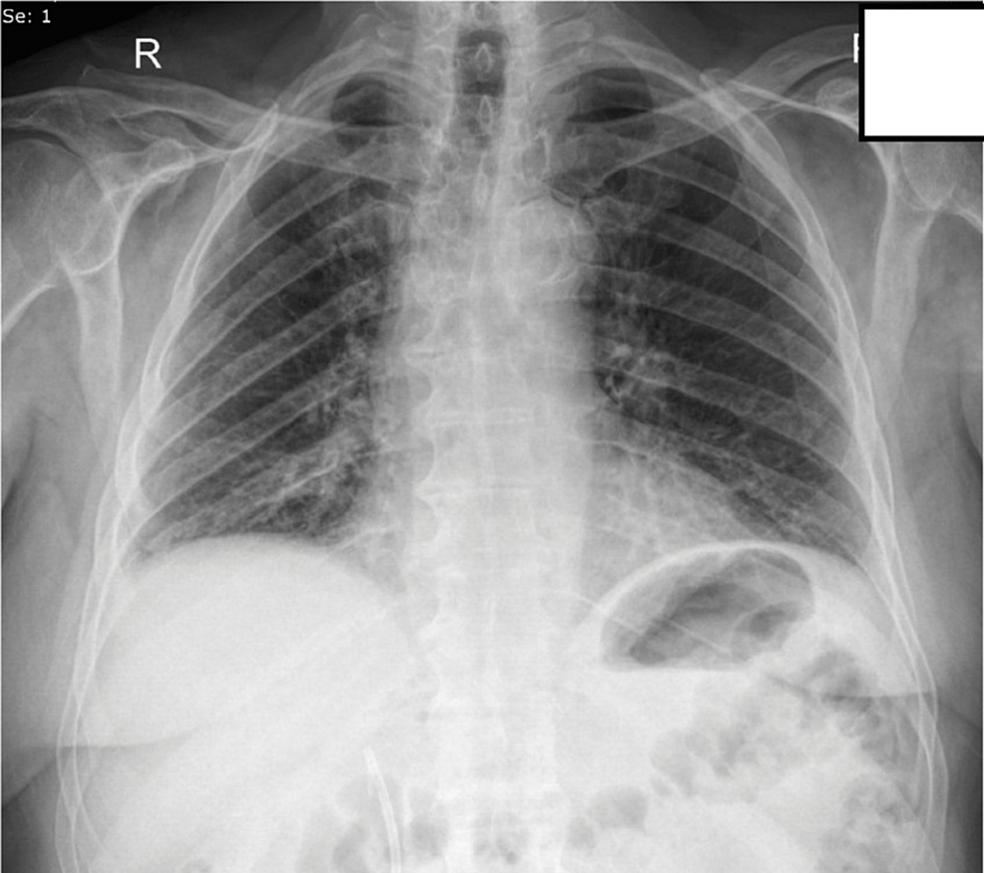
Chest X-ray one month post-discharge No pathological radiographic findings were noted.

The patient returned one month later for common bile duct stent removal and his clinical and radiological condition was excellent, raising no concerns (Figure 7).

## Discussion

This case demonstrates a rather common clinical presentation for a recently operated patient. Postoperative fever is always a clinical entity requiring vigilance and one should be aware of all of the “four W's.” The mnemonic sums up the most common causes: pulmonary causes - “wind”; urinary tract infections - “water”; surgical site infections - “wound”; iatrogenic causes - “what did we do?” PE is considered one of the probable pulmonary causes, as well as one related to increased morbidity and even mortality. So, in this manner, it was correctly taken into consideration early in the diagnostic process [11].

Since, the diagnosis could not be set up front, given the inability to perform a CTPA, the patient had to be treated as there was more than one cause, which justifies both anticoagulants administration, as well as broad-spectrum antibiotics administration. One could question this strategy, but since our patient was in the immediate postoperative period, it was considered optimal to initiate treatment as in the case of PE, awaiting confirmation from the CTPA [4]. During the period between admission and the time the CTPA was performed, the patient was hemodynamically stable with unaltered clinical presentation and afebrile. His clinical status did not raise any concerns to assume that any major event had occurred during hospitalization.

After ruling out PE, the treatment was modified. The main focus was to investigate the subcapsular lesion, which was later proved to be a biloma secondary to intraparenchymal gallbladder rupture. The patient had no known history of gallbladder lithiasis and was asymptomatic prior to this event. Given that liver function tests, total and direct bilirubin, gamma-glutamyl transferase, and alkaline phosphatase were within normal range, and the patency of the common bile duct was secured with stenting, the multidisciplinary team chose to prioritize surgery and then common bile duct stones removal followed [12].

The surgical team chose the open approach over laparoscopy. Since almost two weeks passed from the incidence till surgery, the possibility that the biloma had turned into an abscess was considered high. In addition, due to the increased size of the biloma, an open approach was preferred to have better exposure and visualization. This enhanced to safely perform the cholecystectomy, adequate biloma clearance, and thorough check for potential bile leaks.

This case could be easily considered as a case of spontaneous biloma secondary to gallbladder rupture. In the literature, there are cases of bilomas in patients who had previously undergone cholecystectomies, so bile leak from the biliary tree was the most likely cause [13,14]. One should highlight the fact that in one case, the patient presented with two postoperative complications, i.e., biloma and PE [14].

In our case, the patient did not have signs of acute gallbladder inflammation or tenderness in the right upper quadrant beforehand. The patient had a known history of coronary disease and the vasculopathy could be present in other vessels throughout the body. Abdominal CT revealed extensive atherosclerotic lesions in the patient’s arteries, which could affect blood flow and cause microischemic angiopathy. This finding, in addition to chronic cholecystitis alterations, could result in a thinner gallbladder wall, more susceptible to rupture. Choledocholithiasis itself could have played an important role in the event of gallbladder rupture. The presence of gallstones within the common bile duct could increase the intraductal pressure and therefore compromise the more vulnerable parts of the biliary tree [10].

Upon further investigating other risk factors that led to this event, we found out that for the needs of the orthopedic surgery, the patient received spinal anesthesia. A solution of ropivacaine with morphine was administered to the patient. In the literature, the relation between morphine administration and the sphincter of Oddi constriction is well-defined. This constriction causes an increase in pressure in the biliary tract [15]. Besides its intravenous use, other forms of administration have been also associated with an increase in Oddi sphincter pressure [16]. In our case, the patient received intrathecal anesthesia, using 2.4 mg of ropivacaine and 0.1 milligram of morphine. The used dosage of morphine was within the range, set by already published studies [17]. As an anesthetic commonly used in various operations, this low dosage of intrathecal morphine is equivalent to the effect of multiple milligrams of intravenously administered morphine, even more than 5 mg [18].

One might argue that the intrathecal morphine administration and thus bypassing the blood-brain barrier could not have this effect, as the drug is directly administered in the cerebrospinal fluid [19]. On the other hand, one matter, that is not very well studied, has recently emerged. A recent study has highlighted the event of clearance of various substances from the CSF to blood [20]. Data are still limited and more studies are required to prove this hypothesis, although there are some indications toward this point.

## Conclusions

The clinical presentation of a patient may be misleading. History of surgical operation should broaden the differential diagnosis and the physicians should look carefully for a diagnosis, different than the obvious. In our case, the presumed diagnosis was PE, but it was not considered definite until proven. Intraparenchymal gallbladder rupture and subsequent biloma was a rather unexpected diagnosis. Drug administration in the perioperative period should be examined thoroughly because this iatrogenic intervention altered an otherwise stable condition. The use of morphine for anesthesia caused a chain of events to reveal the unknown cholelithiasis, in a form vastly different than the expected.
